# Lung recruitment state during induction of general anaesthesia in a prospective observational clinical study in patients without and with obesity

**DOI:** 10.1038/s41598-025-91217-3

**Published:** 2025-03-21

**Authors:** Silke Borgmann, Kim Linz, Johannes Schmidt, Sara Lozano-Zahonero, Christin Wenzel, Sashko Spassov, Stefan Schumann

**Affiliations:** 1 Department of Anesthesiology and Critical Care, Medical Center - University of Freiburg, Freiburg, Germany; 2 Faculty of Medicine, University of Freiburg, Freiburg, Germany

**Keywords:** Electrical impedance tomography, Induction of anaesthesia, Loss of end-expiratory lung volume, Ventilation distribution, Ventilation inhomogeneity, Medical research, Clinical trials

## Abstract

We investigated lung aeration during preoxygenation, mask ventilation, ventilation via endotracheal tube, and the two apnoeic phases in-between. Using electrical impedance tomography we assessed global inhomogeneity, ventral-to-dorsal ventilation distribution, the area of ventilated lung and end-expiratory lung volume loss. Global inhomogeneity was increased after the apnoeic phases (non-obese: 25%, obese: 66%, p<0.005 for both) and re-improved with the first breaths of mechanical ventilation (non-obese) or during mask ventilation only (obese). Ventral ventilation increased after the first (non-obese: 52%, obese: 36%) and second apnoeic phase (non-obese: 46%, obese: 36%) compared to spontaneous breathing (all p<0.005). Ventral ventilation was highest in the first eight breaths following the second apnoeic phase in non-obese patients and in the first breath during mask ventilation in patients with obesity. The area of ventilated lung was smallest during the first or first eight breaths following each apnoeic phase in both patient groups. The decrease of end-expiratory lung volume was more pronounced during the first (non-obese: 411 [95%CI 273, 549] ml, obese: 417 [95%CI 325, 509] ml) compared to the second apnoeic phase (non-obese: 239 [95%CI 166, 312] ml, obese: 285 [95%CI 188, 382] ml, p<0.02 for all cases). We conclude that lung derecruitment occurs during the apnoeic phases of anaesthesia induction and resolves partly with subsequent mechanical ventilation.

## Introduction

General anaesthesia and subsequent mechanical ventilation are essential components of perioperative medicine. However, induction of general anaesthesia and mechanical ventilation are related to inhomogeneous distribution of ventilation, redistribution of tidal volume from dorsal to ventral regions, regional over-inflation and lung derecruitment^1–5^. This may contribute to post-operative pulmonary complications^6–8^. Previous studies have shown that collapsed lung areas can be observed as soon as 5 - 10 minutes after induction of anaesthesia^9–12^. These changes in the respiratory system may result from the unphysiological nature of mechanical ventilation. The induction of general anaesthesia is a multi-stage procedure, typically involving preoxygenation, an apnoeic phase caused by the anaesthetic drugs that suppress the breathing drive, subsequent ventilation via face mask, a second apnoeic phase during which endotracheal intubation is conducted and mechanical ventilation after placement of the tracheal tube. However, the contribution of the different stages of anaesthesia induction on the dynamics of lung derecruitment is unclear. We addressed this question by investigating the aeration of the lung throughout the entire process of anaesthesia induction in patients without and patients with obesity. Therefore, we utilized electrical impedance tomography (EIT) to continuously monitor regional lung ventilation from spontaneous breathing during preoxygenation to established mechanical ventilation after endotracheal intubation.

## Methods

Ethical approval for this prospective study was obtained from the Ethics Committee of the University of Freiburg (Freiburg, Germany, file number 262/17, May 2017). The study was registered in the WHO-listed German register for clinical trials (DRKS00012581, June 2017) prior to inclusion of the first patient. This study was conducted in accordance with the applicable guidelines and regulations, including the revised version of the Declaration of Helsinki of the World Medical Association, the applicable federal and state laws, and the medical professional regulations of the Federal Republic of Germany. Written informed consent was obtained from every patient before participation in the study. All patients were enrolled at the Medical Center of the University of Freiburg between June and December 2017.

### Patient population

Patients aged 18 years or older with ASA physical status less or equal to III scheduled for elective surgery were eligible for inclusion in the study. Patients without obesity required a body mass index (BMI) below or equal than 30 kg $\text {m}^{-2}$, and were scheduled for elective general surgery in our central operating facility. Patients with obesity required a BMI above 30 kg $\text {m}^{-2}$, and were scheduled for elective bariatric surgery. Exclusion criteria were pregnancy, suspected difficult airway, active implants (e.g. pacemaker or cardioverter-defibrillator) and a known history of pulmonary disease or indication for induction of anaesthesia without mask ventilation (rapid sequence anaesthesia induction).

### Preparation of patients

Patient preparation was conducted prior to preoxygenation, while the patients were awake and in a sitting position. An electrode belt (Dräger Medical, Lübeck, Germany) for electrical impedance tomography measurements (PulmoVista 500, Dräger) was positioned between the 4th and 5th intercostal space. Routine monitoring was established, including electrocardiography, pulse oximetry, and non-invasive blood pressure measurement. Additionally, bispectral index (BIS) electrodes (Medtronic, Minneapolis, USA) were placed on the patient’s forehead for monitoring anaesthesia depth, and a mechanomyograph (ToFscan, Dräger) was applied. Subsequently, patients were asked to lie down in supine position. In accordance with our clinical guidelines, patients with obesity were kept with upper body elevated at $30^{\circ }$ throughout the entire induction sequence.

### General anaesthesia

Preoxygenation was continued until a fraction of expired oxygen $\ge$ 0.8 was achieved. Anaesthesia was induced by administration of 0.3–0.5 μg $\text {kg}^{-1}$ sufentanil (Sufenta mite, Janssen-Cilag, Neuss, Germany) followed by the intravenous administration of propofol (Propofol 1%, MCT Fresenius Kabi, Bad Homburg vor der Höhe, Germany). For patients without obesity, target controlled infusion of propofol with an effective concentration of 6–9 μg $\text {ml}^{-1}$ (Injectomat $\circledR$TIVA Agilia, Fresenius Kabi, AG, Bad Homburg vor der Höhe, Deutschland) was established. Patients with obesity received a bolus of propofol ranging from 1.0 to 2.5 mg $\text {kg}^{-1}$. Subsequently, 0.1–0.15 mg $\text {kg}^{-1}$ predicted body weight cisatracurium (NIMBEX, GlaxoSmithKline, Brentford, United Kingdom) was administered to facilitate endotracheal intubation. During subsequent ventilation (Fabius Tiro, Dräger) via face mask positive end-expiratory pressure (PEEP) was set to 3 $\text {cmH}_{2}$O in patients without obesity, and to 5 $\text {cmH}_{2}$O in patients with obesity. After endotracheal intubation, all patients were ventilated in volume controlled mode (Fabius Tiro, Dräger) with a tidal volume of 6–8 ml $\text {kg}^{-1}$ predicted body weight. The ventilation frequency was adjusted to maintain an end-tidal carbon dioxide partial pressure between 35 and 45 mmHg. For patients without obesity, PEEP was set to 7 $\text {cmH}_{2}$O and to 10 $\text {cmH}_{2}$O for patients with obesity.

### Data collection

Data collection commenced with preoxygenation and concluded after at least one minute of stable mechanical ventilation following endotracheal intubation. Respiratory data were continuously recorded at a rate of 100 Hz from the anaesthesia machine through dedicated software (Proto4Service, Dräger). Simultaneously, EIT data were continuously recorded at a rate of 20 images per second.

### Data processing

Data analyses were performed using custom code developed in Matlab (R2020b, The MathWorks, Natick, USA). Functional tidal images were calculated for the three non-apnoeic measurement periods as described earlier^13^. In brief, impedance images were reconstructed using the EIT Data Analysis Tool 6.1 (Dräger). Within these reconstructed images, those corresponding to the beginning and the end of inspiration were identified. For each breath, the image corresponding to the beginning of inspiration was subtracted from the image at the end of inspiration. The resulting tidal images of the breaths were then averaged resulting in functional tidal images for each non-apnoeic phase. To assess the functional tidal images, we individually defined the lung area for each patient using the IDEAL method^14^ on functional tidal images during spontaneous breathing in the preoxygenation phase. Briefly, for each image, the maximum tidal impedance change was determined. Subsequently, the lung area of each patient was defined as the set of all pixels with intratidal impedance changes larger than 20% of the respective maximum value.

To assess the aeration and recruitment state of the lung, four parameters of regional ventilation were determined: The global inhomogeneity index was calculated to quantify the inhomogeneity of the ventilation distribution within the lung^15^. A larger global inhomogeneity index reflects a more inhomogeneous ventilation pattern. As an estimate for ventral to dorsal ventilation distribution, tidal variation was determined as the percentage of ventilation present in ventral and dorsal parts of the lung^13^. To achieve this, we divided the functional tidal images in half along the central horizontal axis, defining ventral and dorsal regions of interest accordingly^13^. Thus, the venrtal region is defined as the upper half of a functional image, the dorsal region as the lower half of it. To quantify the changes in the aerated lung region, we determined the area of ventilated lung as the number of ventilated pixels of a functional tidal image during the respective phases of mask ventilation and ventilation via endotracheal tube again applying the IDEAL method. These areas were then compared to the area of ventilated lung during spontaneous breathing. Loss of end-expiratory lung volume was calculated as described earlier^16^. In brief, the global impedance curve^13^ was used to calculate the average of the impedances at the start of inspiration during preoxygenation, mask ventilation and ventilation via endotracheal tube. The differences between these averages are associated with the loss of end-expiratory lung volume. Similarly, the loss of end-expiratory lung volume during the apnoeic phases was assessed: the impedance differences between beginning and end of the apnoeic phases were calculated. All impedance differences were than scaled to units of ml adhering to Grivans et al.^16^. For the phases of mask ventilation and ventilation via endotracheal tube, analyses were performed for three different breath ranges to better examine the dynamic changes in the aeration state of the lung after the apnoeic phases: (a) the first complete breath after apnoea, (b) the average of the first eight consecutive breaths of the respective ventilation phase and (c) the average over all available breaths within each ventilation phase were used for analysis.

### Statistics

As no comparable previous data were available, we chose an explorative approach omitting a sample size calculation. We chose a sample size of fifteen patients per group as we expected to be able to observe the characteristic of lung derecruitment with this sample size. However, we did conduct a post hoc power calculation using the changes in tidal variation as a primary endpoint. We chose this parameter, since it has been investigated using EIT a short time after intubation, showing a redistribution of air from dorsal to ventral areas. When using the observed effect sizes (non-obese: 0.85, obese: 0.95) and an $\alpha$-level of 5% in a model of repeated measures ANOVA with a nonsphericity correction of 0.5 to account for the reduced degrees of freedom introduced by the nested nature of the analysed breath ranges, the estimated power was 99.8% for patients without obesity and 99.9% for patients with obesity. Due to the high power, we did choose to conduct hypothesis testing using a repeated measures ANOVA to investigate our data followed by a paired t-test for post-hoc analysis. Data are presented as mean and the 95% confidence interval in square brackets unless stated otherwise. We assumed comparisons of variables showed a significant difference if the calculated p-value was smaller than 5%.

## Results

**Table 1:** Patient charactaristics. Summary of the relevant data and anaesthesia related parameters of all patients included into this study. For both, the patients without and with obesity values are given as mean (SD) where applicable. BMI is the abbreviation for body mass index, BIS the abbrevation for bispectral index.

	Non-obese patients	Obese patients
	(n = 14)	(n = 15)
Sex [female]	2	12
Height [cm]	174 (11)	169 (10)
Weight [kg]	77 (11)	133 (21)
BMI [kg $\text {m}^{2}$]	25 (3)	47 (8)
Age [years]	51 (15)	43 (13)
ASA status [I/II/III]	4/6/4	–/8/7
Sufentanil [μg]	28.7 (4.4)	28.0 (6.5)
Propofol [mg $\text {ml}^{-1}$]	8.4 (1.4)	
Propofol [mg]		284 (80)
Cis-Atracurium [mg]	8.9 (1.4)	9.0 (1.2)
BIS	29 (6)	28 (9)

**Fig. 1:**
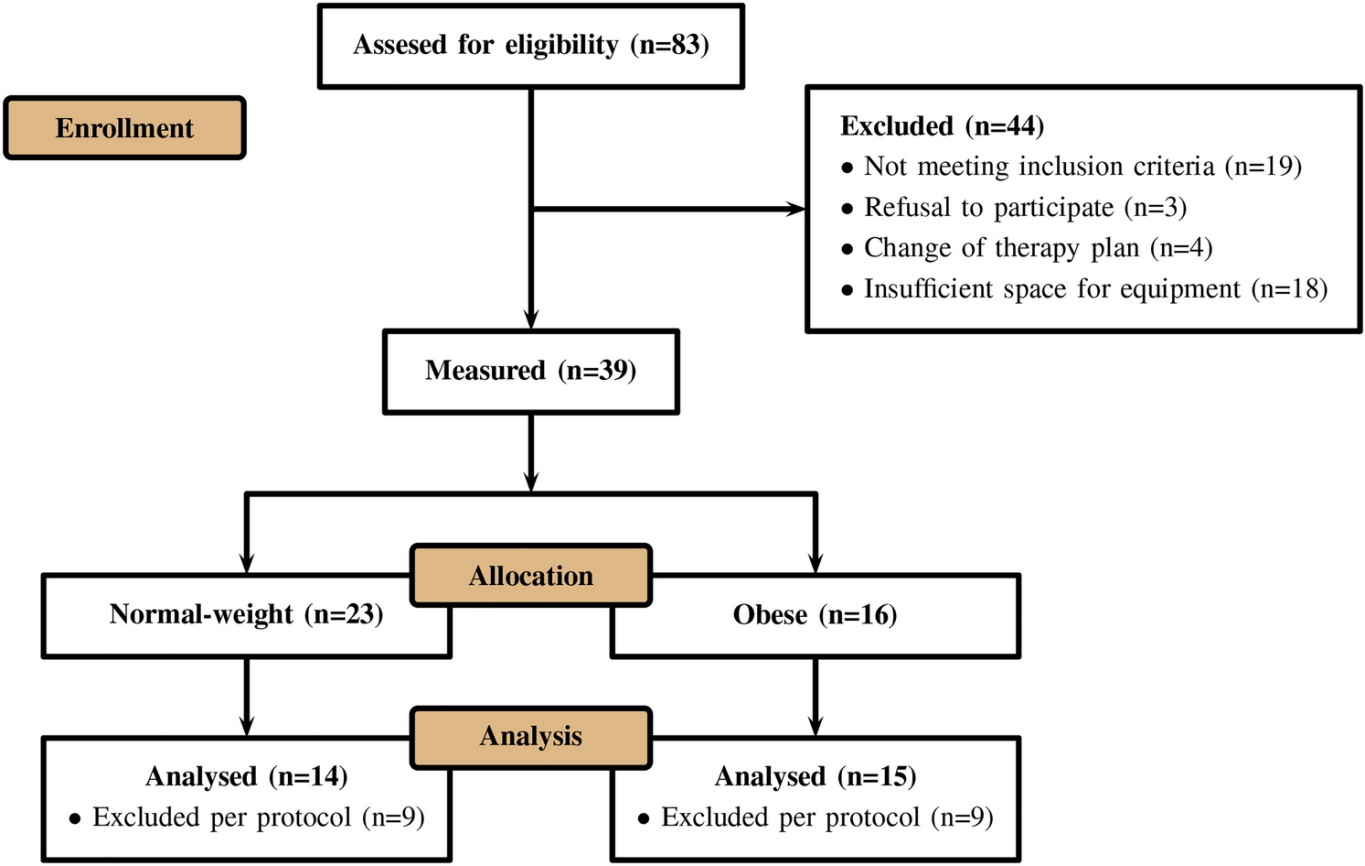
CONSORT flow chart. Illustration of the process from enrollment of patients to analysis of data sets thereby detailing exclusion of seemingly eligible patients.

**Fig. 2:**
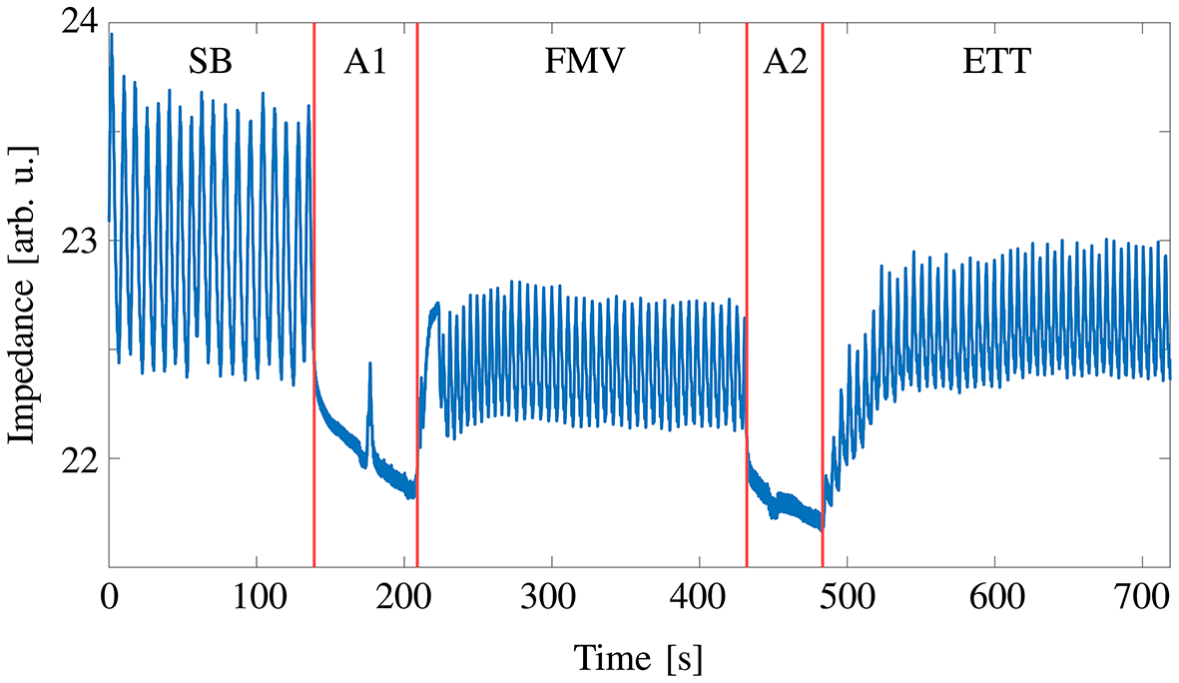
Global impedance curve. Representative global impedance curve derived from an exemplary EIT recording of a patient with obesity, illustrating the different phases as per the study protocol. Initially, patients are breathing spontaneously (SB). During this phase, anaesthesia is induced, leading to an apnoea phase (A1). The apnoeic phase concludes with the initiation of mask ventilation (FMV). Following FMV, a second apnoea phase (A2) ensues due to the placement of the tracheal tube. Finally, ventilation is commenced via tracheal tube (ETT). Red vertical lines have been added to aid visual clarity and separate the distinct phases.

Eighty three patients were screened for eligibility. Nineteen did not meet the inclusion criteria, three refused to participate, for four patients the therapy plan changed and in 18 cases there was not enough space for the measurement equipment (Fig. 1). Measurements were performed in 23 patients without obesity and 16 patients with obesity. Data from 8 patients without obesity and from one patient with obesity had to be excluded due to corrupted data recordings. One patient without obesity did not fit our inclusion criterion after BMI was calculated retrospectively. This patient was very muscular, leading to a BMI larger than 30 kg $\text {m}^{-2}$ without actually appearing obese. A summary of the patients charactarisitcs is given in Table 1. Haemodynamic and respiratory variables were in the expected physiological range for patients without and with obesity (Tables 1 and 2). The five phases of induction of general anaesthesia were clearly distinguishable in the global impedance curve (Fig. 2). The phases of tidal breathing (spontaneous breathing during preoxygenation, mask ventilation and ventilation via endotracheal tube) showed clear tidal impedance changes. By contrast, during the apnoeic phases global impedance decreased while no tidal impedance changes occurred.

**Table 2:** Haemodynamic and respiratory data. Haemodynamic and respiratory variables for patients without and with obesity during spontaneous breathing, mask ventilation and ventilation via endotracheal tube. All values are given as mean (SD). $\text {BP}_{\text{sys}}$ systolic blood pressure, $\text {BP}_{\text{dia}}$ diastolic blood pressure, $\text {BP}_{\text{mean}}$ mean arterial pressure, HR heart rate, $\text {SpO}_{2}$ peripheral oxygen saturation, $\text {FiO}_{2}$ fraction of inspired oxygen, $\text {FeO}_{2}$ fraction of expired oxygen, $\text {PetCO}_{2}$ end-tidal carbon dioxide partial pressure, PIP peak inspiratory pressure, VT tidal volume, I:E duration of inspiration to duration of expiration. Values are given as mean (SD).

	Non-obese patients			Obese patients		
	Spontaneous breathing	Ventilation via face mask	Ventilation via endotracheal tube	Spontaneous breathing	Ventilation via face mask	Ventilation via endotracheal tube
$\text {BP}_{\text{sys}}$ [mmHg]	141 (13)	111 (23)	112 (16)	152 (15)	118 (18)	125 (20)
$\text {BP}_{\text{dia}}$ [mmHg]	84 (9)	64 (14)	68 (16)	81 (13)	66 (14)	68 (11)
$\text {BP}_{\text{mean}}$ [mmHg]	108 (11)	83 (15)	86 (16)	108 (15)	89 (17)	92 (14)
HR [$\text {min}^{-1}$]	77 (18)	62 (14)	66 (14)	79 (16)	65 (14)	68 (15)
$\text {SPO}_{2}$ [%]	99 (2)	99 (1)	99 (1)	99 (3)	99 (1)	99 (3)
$\text {FiO}_{2}$ [%]	96 (5)	96 (5)	87 (10)	97 (3)	96 (6)	78 (14)
$\text {FeO}_{2}$ [%]	74 (16)	90 (7)	84 (8)	82 (9)	88 (8)	72 (14)
$\text {PetCO}_{2}$ [mmHg]	24 (9)	33 (6)	40 (5)	33 (7)	34 (7)	39 (4)
PIP [mmHg]	–	13.3 (2.2)	18.4 (2.5)	–	17.1 (2.4)	25.7 (2.6)
VT [ml]	–	670 (193)	534 (77)	–	529 (147)	466 (75)
I:E ratio	–	1.8 (0.2)	1.9 (0.3)	–	1.8 (0.1)	2.0 (0.1)
RR [$\text {min}^{-1}$]	–	10 (2)	11 (2)	–	11 (2)	11 (2)
Peak pressure [mbar]	–	13 (2)	18 (2)	–	17 (2)	26 (3)
Plateau pressure [mbar]	–	13.6 (2.3)	16.1 (2.2)	–	17.4 (2.5)	22.3 (2.4)

### Patients without obesity

**Fig. 3:**
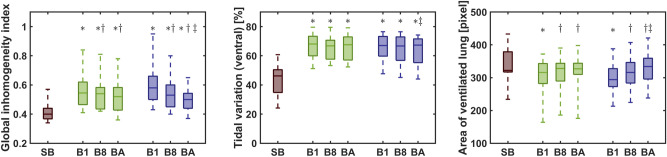
Overview of the studied parameters in patients without obesity. Inhomogeneity (left), ventilation distribution (center) and area of the ventilated lung (right) for patients without obesity. Each variable is presented for the average over all breaths of spontaneous breathing (SB), for the first breath (B1), for the average over the first eight breaths (B8) as well as for the average over all breaths (BA) from each mechanical ventilation phase. Green bars mark the phase of mask ventilation and blue bars the phase of ventilation via endotracheal tube, respectively. $*$ marks a significant difference to SB, $\dagger$ marks a significant difference to the first breath of the mask ventilation phase/phase of ventilation via endotracheal tube, $\ddagger$ marks a difference to the first eight breaths of the mask ventilation phase/phase of ventilation via endotracheal tube.

**Fig. 4:**
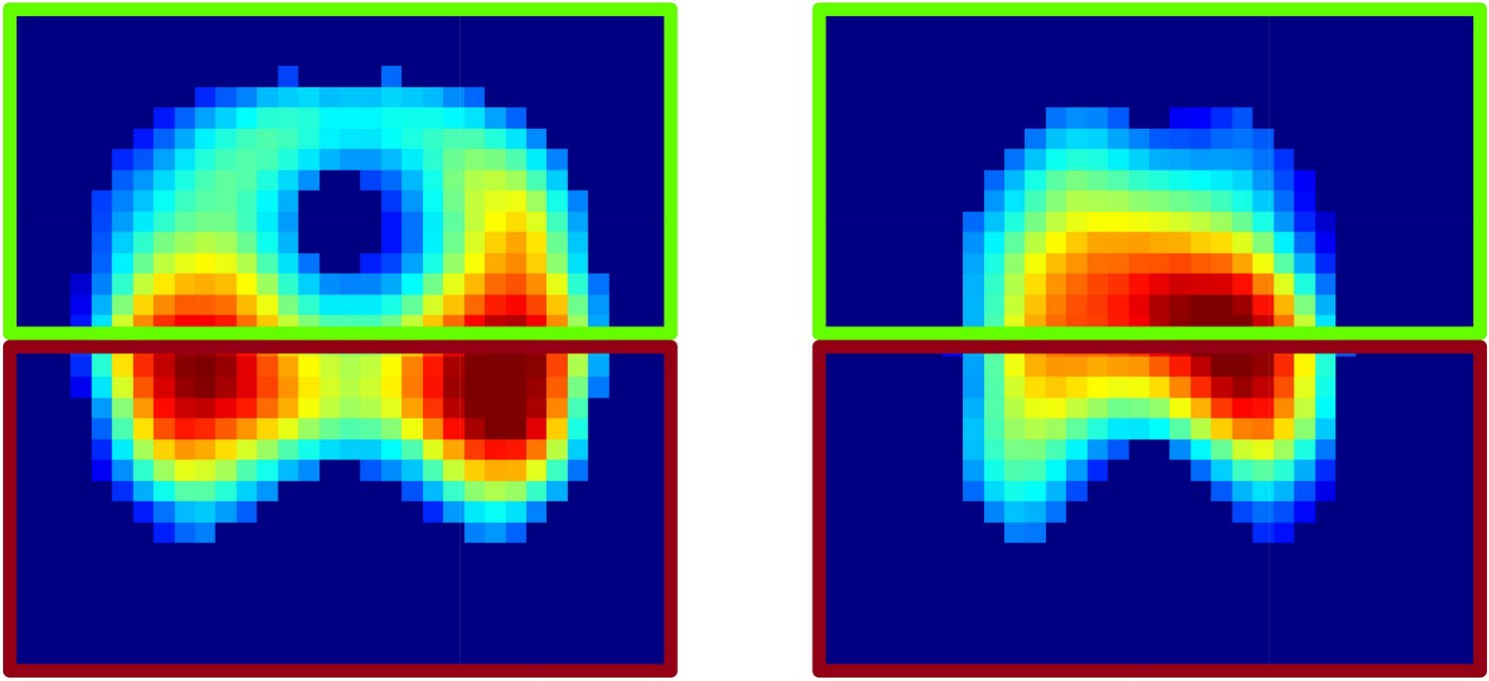
Functional tidal images for both patient groups. Distribution of regional ventilation during spontaneous breathing in a representative patient without (left) and a patient with obesity (right). Warmer colours represent higher impedance values and hence more ventilation, colder colours areas with little to no ventilation. The green frame marks the ventral area, the red frame the dorsal area.

Global inhomogeneity indices were lower during the initial spontaneous breathing phase compared to both mask ventilation and ventilation via endotracheal tube (all p<0.005, Fig. 3). The global inhomogeneity indices were consistently highest for the first breath following an apnoeic phase and decreased with an increasing number of breaths included in the analysis (Fig. 3). This decrease was significant during mask ventilation (all p<0.001), but not during ventilation via endotracheal tube (all p>0.07). During spontaneous breathing regional ventilation was predominantly located in the dorsal areas (Figs. 3 and 4). Beginning with the first breath of mask ventilation and ventilation via endotracheal tube, the percentage of regional ventilation was shifted towards the ventral areas (p<0.0001 for all three ranges of included breaths). The share of ventral ventilation did not change in the course of mask ventilation (p>0.5). During ventilation via endotracheal tube, the share of ventral ventilation was highest for the first eight breaths and decreased with increasing number of breaths included in the analysis (Fig. 3). There were no differences in area of ventilated lung between spontaneous breathing and the two mechanical ventilation phases. The area of ventilated lung was smaller during the first breath and the first eight breaths compared to the entire phase of ventilation via endotracheal tube (p = 0.007 and p = 0.0048, Fig. 3). During the first apnoeic phase 411 [273, 549] ml lung volume were lost, during the second apnoeic phase 239 [166, 312] ml were lost (p = 0.018). More end-expiratory volume was lost from preoxygenation to mask ventilation (275 [169, 381] ml) compared to preoxygenation and ventilation via endotracheal tube (88 [− 48, 223] ml, p = 0.042).

### Patients with obesity

**Fig. 5:**
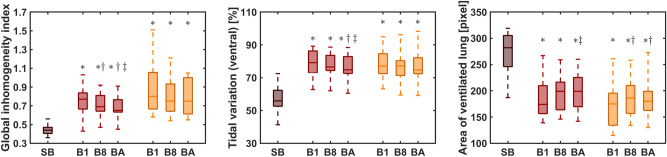
Overview of the studied parameters in patients with obesity. Inhomogeneity (left), ventilation distribution (center) and area of ventilated lung (right) for patients with obesity. Each variable is presented for the whole phase of spontaneous breathing (SB), for the first breath (B1), for the average over the first eight breaths (B8) as well as for the average over all breaths (BA) from each mechanical ventilation phase. Red bars mark the phase of mask ventilation and orange bars the phase of ventilation via endotracheal tube respectively. $*$ marks a significant difference to SB, $\dagger$ marks a significant difference to the first breath of the mask ventilation phase/phase of ventilation via endotracheal tube, $\ddagger$ marks a difference to the first eight breaths of the mask ventilation phase/phase of ventilation via endotracheal tube.

The global inhomogeneity indices were lower during spontaneous breathing compared to both mask ventilation and ventilation via endotracheal tube (all p<0.005, Fig. 5). The global inhomogeneity indices were consistently highest in the first breath following an apnoeic phase and decreased with the number of breaths included in the analysis (Fig. 5). This decrease was significant during mask ventilation (all p<0.003) but not during ventilation via endotracheal tube (all p>0.125). During spontaneous breathing regional ventilation was predominantly located in the ventral areas (Figs. 4 and 5). However, during both mask ventilation and ventilation via endotracheal tube, the pre-existing ventral shift was further increased (all p<0.0001 for all three ranges of included breaths). Moreover, the shift of ventilation towards the ventral areas was higher for the first breath and during the first eight breaths compared to all breaths during mask ventilation (all p<0.03). In contrast, no differences between all breaths and the first or the first eight breaths were observed during ventilation via endotracheal tube (p = 0.1782 and p$=$0.7877 respectively, Fig. 5). The area of ventilated lung was significantly smaller during both phases of mechanical ventilation than during spontaneous breathing (all p<0.0001, Fig. 4). Additionally, the area of ventilated lung was smaller during the first eight breaths than during the entire phase of mask ventilation (p$=$0.031) but not during the first breath and the entire phase of mask ventilation (p$=$0.08). During ventilation via endotracheal tube, the area of ventilated lung remained unchanged for all ranges of included breaths (all p>0.06, Fig. 5). During the first apnoeic phase 417 [325, 509] ml lung volume were lost, during the second apnoeic phase 285 [188, 382] ml were lost (p = 0.02). The loss in end-expiratory volume from preoxygenation to mask ventilation (145 [44, 245] ml) was comparable to the loss from preoxygenation to ventilation via endotracheal tube (275 [31, 519] ml, p = 0.348).

## Discussion

Our main results are that lung inhomogeneity, shifts of ventilation from dependent to independent lung regions, and loss of ventilated lung volume were present immediately after the apnoeic phases during anaesthesia induction in patients without and with obestiy. This indicates that anaesthesia-related derecruitment results rather from the apnoeic phases than from mechanical ventilation. Moreover, lung inhomogeneity and ventilated lung area improved during the first breaths of mechanical ventilation, indicating that mechanical ventilation may re-recruit lost lung tissue. A vivid illustration of evolution of the global inhomogeneity, the shares of ventral ventilation and the area of ventilated lung during the first eight breaths of both phases of mechanical ventilation is provided in the supplement (Figs. S1 and S2, online Supporting Information). The resolvement of the derecruiting effects of the apnoeic phases can be clearly seen in this representation.

It should be noted that, due to the exploratory nature of our investigation, we did not perform an a priori sample size calculation. Therefore, the results of the hypothesis testing should be interpreted with caution. However, due to the strong results of the power analysis conducted, we have decided to report p-values along with confidence intervals to enhance the interpretability of our findings.

To the best of our knowledge, our study is the first to use EIT to continuously monitor the whole procedure of induction of anaesthesia in adult patients without and with obestiy. The induction of anaesthesia has only been investigated continuously in children before^17^. However, less homogeneous ventilation, shifts of ventilation from dependent to independent regions and atelectasis formation in both patients without and with obestiy have already been reported using EIT or CT scans^9–12,18–21^ albeit well after endotracheal intubation. Our findings not only confirmed earlier findings, but demonstrated that the onset of all these phenomena was either during or immediately after the apnoeic phases which seem to exert a notable influence on the recruitment state of the lung. This is best illustrated by the decrease of global impedance during the apnoeic phases (Fig. 2) showing a prominent loss of end-expiratory lung volume^16^. Patients lost recruited lung volume of about 400 ml during the first and about 250 ml during the second apnoeic phase. Also lung inhomogeneity and shifts of ventilation to ventral areas characterized by the poorest outcomes in the first breaths. Consequently, we feel justified to deduce that the apnoeic phases are a main contributor to lung derecruitment during induction of anaesthesia. A limitation of our study is the incomparability of patients without and with obestiy, since both groups had different induction procedures and a different gender distribution. However, while we did not primarily focus on comparisons between patients without and with obestiy beyond a descriptive approach, characteristic differences between both patient groups became apparent: We interpret particularly the decrease of the area of ventilated lung as a measure for derecruitment. Already during spontaneous breathing the area of ventilated lung in patients with obesity was smaller than in patients without obesity. This might suggest a presence of derecruited lung areas in patients with obesity even during spontaneous breathing, which confirms previous findings from CT scans^20^. In addition, induction of anaesthesia worsened the aeration state of the lung in patients with obesity more than in patients without obesity. This was reflected in higher increases of lung inhomogeneity, higher ventral shift of ventilation and a higher loss of ventilated lung area. This is particularly noteworthy, considering that patients with obesity were positioned with an elevated upper body and received higher PEEP than patients without obesity, with the intention to counteract such lung impairment. For patients without obesity, we found further derecruitment after the second apnoeic phase. This might suggest to either shorten the preceding apnoeic phase or, considering the potential recruiting effect of mask ventilation with PEEP^22^ in between, to prolong the mask ventilation phase until a steady state in aeration is observed. However, this would necessitate mechanical ventilation and EIT monitoring. Further studies would be needed to assess such an approach. The evolution of derecruited volume was different in patients with obesity. Even though losses of lung volume during both apnoeic phases were comparable to patients without obesity, PEEP after the second apnoeic phase could only partly counteract previous derecruitment. This is especially interesting since PEEP doubled from mask ventilation to ventilation via endotracheal tube. Moreover, lung homogeneity, shares of ventral ventilation and area of ventilated lung resolved only marginally, if at all, during the mechanical ventilation phases in patients with obesity. In other words, apnoea related derecruitment persists at least till the end of anaesthesia induction. Since further our findings indicate that for patients with obesity a PEEP of 5 $\text {cmH}_{2}$O during mask ventilation and a PEEP of 10 $\text {cmH}_{2}$O during ventilation via endotracheal tube was not sufficient to restore the lung aeration state of spontaneous breathing, this opens the question for measures to counteract this. The progression of impedance loss during apnoea (Fig. 2) may indicate that the duration of the apnoeic phase plays a major role for derecruitment and one may deduce that the duration of the apnoeic phases should be minimized. One way to achieve this would be rapid sequence induction^23^. However, rapid sequence induction bears risks itself and is not applicable for all patients, especially not for patients with presumed difficult airway for which obesity is a risk factor^24^. The application of a lung recruitment manoeuvre or the application of mask continuous pressure ventilation during anaesthesia induction might be an applicable means. The latter has been shown to improve oxygenation and prevent atelectasis formation during the induction of general anesthesia^12,25^.

## Conclusion

In conclusion, we have shown that ventilation inhomogeneity, redistribution of ventilation from dorsal to ventral areas, and lung derecruitment, follow the apnoeic phases during anaesthesia induction in patients without and with obesity. Mechanical ventilation led to improvements of the apnoea related lung derecruitment. Strategies to minimize the duration of apnoeic phases or recruiting the lung after the apnoeic phases need to be investigated. Especially patients with obesity may benefit from further optimizations of the process of anaesthesia induction.

## Supplementary Information

Supplementary Figure 1.

Supplementary Figure 2.

## Data Availability

The datasets generated during and/or analysed during the current study as well as custom code used in the analyses are available from the corresponding author upon request.
