# Supplementary material for: Lung recruitment state during induction of general anaesthesia in a prospective observational clinical study in patients without and with obesity
Source: Sci Rep. 2025 Mar 21;15:9773. doi: 10.1038/s41598-025-91217-3 (PMC11928622; doi:10.1038/s41598-025-91217-3)
Supplement: Supplementary file 1 — Supplementary Figure 1. [file 41598_2025_91217_MOESM1_ESM.pdf]

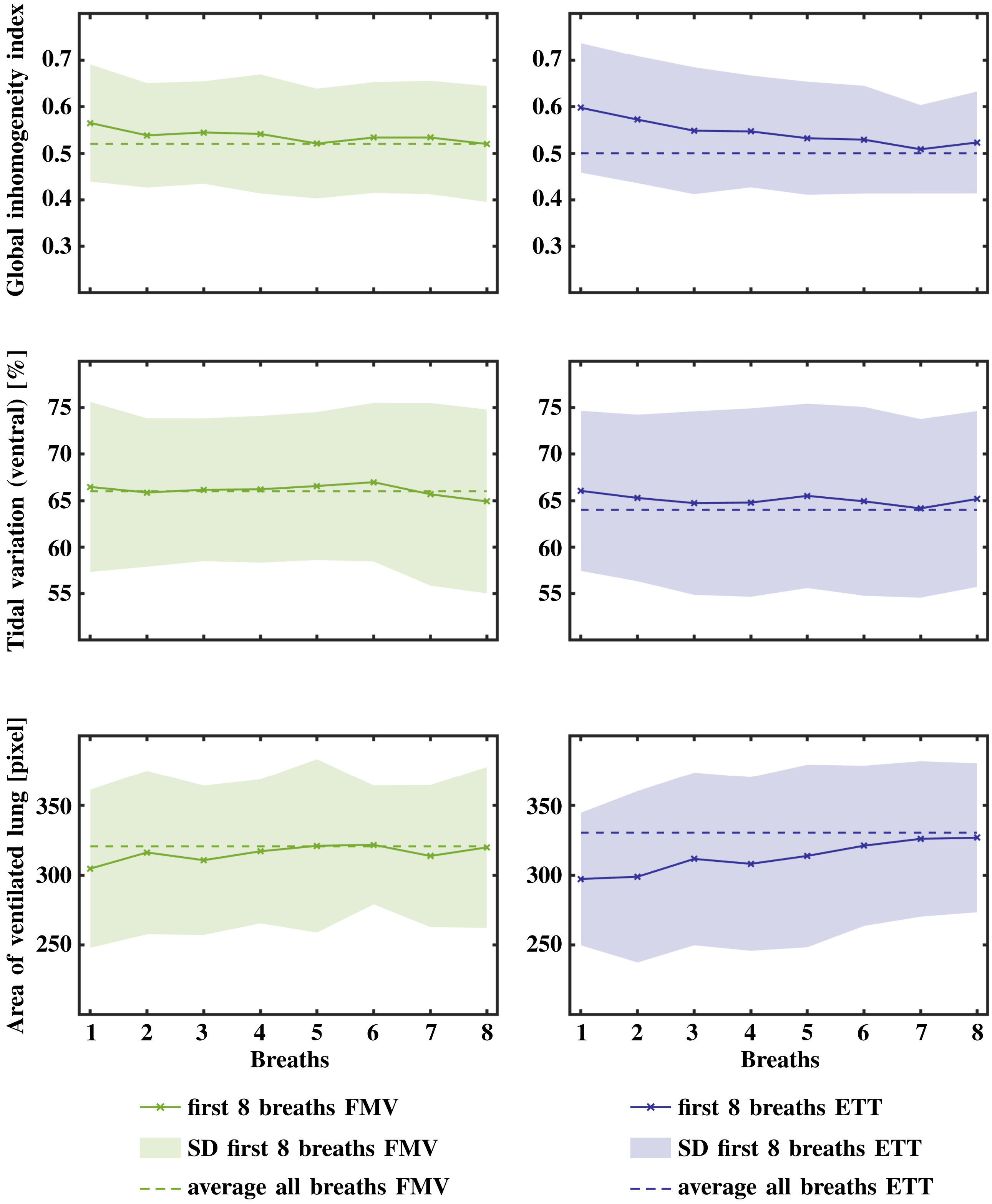

Figure S1. Dynamics of lung derecruitment during mechanical ventilation illustrated by single breaths. The evolution of the global inhomogeneity index, the tidal variation in the ventral part of the lung and the area of ventilated lung during the first eight breaths of each phase of mechanical ventilation is shown in non-obese patients. It can be clearly seen that the changes in each parameter observed during mechanical ventilation are present immediately after the apnoeic phases. The improvement of all parameters during the first eight breaths can be seen in each mechanical ventilation phase. However, this improvement was more pronounced during the invasive ventilation phase. FMV: ventilation via face mask; ETT: ventilation via endotracheal tube.
